# Personalized Pathway Enrichment Map of Putative Cancer Genes from Next Generation Sequencing Data

**DOI:** 10.1371/journal.pone.0037595

**Published:** 2012-05-18

**Authors:** Peilin Jia, Zhongming Zhao

**Affiliations:** 1 Department of Biomedical Informatics, Vanderbilt University School of Medicine, Nashville, Tennessee, United States of America; 2 Department of Psychiatry, Vanderbilt University School of Medicine, Nashville, Tennessee, United States of America; 3 Department of Cancer Biology, Vanderbilt University School of Medicine, Nashville, Tennessee, United States of America; University of California Los Angeles, United States of America

## Abstract

**Background:**

Pathway analysis of a set of genes represents an important area in large-scale *omic* data analysis. However, the application of traditional pathway enrichment methods to next-generation sequencing (NGS) data is prone to several potential biases, including genomic/genetic factors (e.g., the particular disease and gene length) and environmental factors (e.g., personal life-style and frequency and dosage of exposure to mutagens). Therefore, novel methods are urgently needed for these new data types, especially for individual-specific genome data.

**Methodology:**

In this study, we proposed a novel method for the pathway analysis of NGS mutation data by explicitly taking into account the gene-wise mutation rate. We estimated the gene-wise mutation rate based on the individual-specific background mutation rate along with the gene length. Taking the mutation rate as a weight for each gene, our weighted resampling strategy builds the null distribution for each pathway while matching the gene length patterns. The empirical *P* value obtained then provides an adjusted statistical evaluation.

**Principal Findings/Conclusions:**

We demonstrated our weighted resampling method to a lung adenocarcinomas dataset and a glioblastoma dataset, and compared it to other widely applied methods. By explicitly adjusting gene-length, the weighted resampling method performs as well as the standard methods for significant pathways with strong evidence. Importantly, our method could effectively reject many marginally significant pathways detected by standard methods, including several long-gene-based, cancer-unrelated pathways. We further demonstrated that by reducing such biases, pathway crosstalk for each individual and pathway co-mutation map across multiple individuals can be objectively explored and evaluated. This method performs pathway analysis in a sample-centered fashion, and provides an alternative way for accurate analysis of cancer-personalized genomes. It can be extended to other types of genomic data (genotyping and methylation) that have similar bias problems.

## Introduction

In large-scale sequencing studies of cancer genomes, one of the central challenges is to distinguish disease-causing “driver” mutations from “passenger” mutations, and allow the development of targeted therapy and medication. While statistical methods have been under active development to test mutation events at the gene level, the combinatorial occurrence of many genes shows distinguishable patterns. Some well-studied examples include mutually exclusive mutations such as *EGFR* and *KRAS* in lung cancer [1], and *TP53* and *MDM2* in glioblastoma. Most of these mutations were frequently observed in certain focused pathways, e.g., four genes from the EGFR-RAS-RAF signaling pathway, *EGFR*, *KRAS*, *HER2*, and *BRAF*, behave in a mutual exclusive fashion in lung cancer [1], [2]. In addition, the most recent findings of The Cancer Genome Atlas (TCGA) projects strongly suggested the convergence of mutations at the pathway level (e.g., three key pathways in glioblastoma, [3]).These observations promoted an emerging consensus that driver genes could be analyzed at the pathway level and induce more straightforward functional interpretation.

The rapid advance in next-generation sequencing (NGS) technologies has made it possible to sequence individual genomes in a timely and cost-efficient manner. For example, whole genome sequencing can provide a full spectrum of the genetic mutations, including single nucleotide variants (SNVs), short insertions/deletions (indels), copy number variations (CNVs), and structure variants. So far, many individual cancer genomes have been successfully sequenced [4], [5], [6], and even more are expected in the near future. These applications provide valuable sequencing data for individual genomes and make it possible to conduct analysis in a sample-centered way, greatly quickening our steps towards personalized diagnose and medication.

In this work, we aimed to perform a pathway-enrichment test of a group of putative cancer genes detected in individual patients. In contrast to most traditional data types, personalized sequencing data is typically complicated by the following features: (1) the mutated genes are related to one individual and likely differ across multiple individuals; (2) the mutated genes occur at an individual-specific background mutation rate, which could be subject to personal life-style, the frequency and dosage of exposure to mutagens, and the particular disease; and (3) the mutated genes are attributed to gene length under the assumption that mutations evenly occur across the whole genome. Due to these challenges, methods that have been well-studied and widely applied in standard gene set analyses are not directly applicable. For example, a functional enrichment test is an important way to explore the biological functions for a list of genes of interest. Traditionally, the genes of interest are derived through studies of a group of samples, e.g., differentially expressed (DE) genes derived from case/control design, and standard statistical tests such as the hypergeometric test or Fisher's exact test can be performed to test if a gene set (e.g., pathway or functional group) is significantly enriched with DE genes. Notably, a common assumption underlying these tests is that all the genes (corresponding to the balls in an urn) have an equal chance of being selected. However, when applied to NGS data, the mutation unit is genomic DNA, e.g., SNVs or small insertions/deletions (indels), and they are assumed to occur evenly across the genome. In contrast, the analyzing unit of a pathway enrichment test is gene. A bias frequently observed in the process of relating SNVs or indels to genes is that long genes tend to harbor more mutations, as they occupy larger parts of the genome, and thus, long genes tend to have higher chance to be mutated. Therefore, the standard hypergeometric test or Fisher's exact test is no longer applicable to such data types.

The long gene effect has been recognized in NGS mutation data. In the recent work of Wendl et al. [7], to estimate the probability of a pathway being enriched with mutated genes, a brute force way of computing the exact *P* values was described, and a convolution-based approximation strategy was proposed aiming to reduce the computational burden. The gene length bias has also been recognized in RNA sequencing data, in which long transcripts tend to have more reads mapped to them. In the work by Young et al. [8], the authors proposed to fit a probability weighting function and quantitatively estimate the probability of a transcript being selected as DE as a function of its transcript length. The Gene Ontology (GO) enrichment test is then performed based on the estimated probability for each transcript/gene. Notably, the gene length bias appears in many aspects of pathway-related analysis, such as pathway crosstalk within each sample and pathway co-mutation profile across multiple samples [9]. Appropriate adjustment could warrant the accuracy of these analyses.

In this study, we proposed a bias-reducing strategy for pathway enrichment test by taking the background of gene-specific mutation rates. This strategy, namely the weighted resampling method, takes into account gene length to estimate the pathway *P* values and has proved to be computationally efficient. Under the weighted resampling framework, personalized pathway crosstalk could subsequently be explored, revealing the complex interaction at the pathway level. In addition, we showed that with effective reduction of gene length bias, a more functionally relevant co-mutated pathway map could be derived. The work we proposed here will find wide applications in the near future as more personalized sequencing data are expected to be available.

## Materials and Methods

### Datasets

#### Pathway collection

We collected all the pathways from KEGG [10] using the R package ‘org.Hs.eg.db’ (version 2.5.0), in which the KEGG pathways were downloaded as of March 15, 2011. A total of 229 pathways and 5891 genes were involved in this version. To avoid pathways defined for too specific or too general biological processes, we selected those with at least 10 and at most 500 genes, resulting in 213 valid pathways for our subsequent analysis.

#### Lung adenocarcinomas data

The lung cancer dataset was initially reported in Ding et al. [11], in which a total of 188 lung adenocarcinomas samples were sequenced for 623 genes. In summary, 163 samples were observed to have mutations in at least one gene, and 356 genes were observed to have mutation(s) in at least one sample. To ensure the statistical power, we included only those samples having at least 10 mutated genes (Figure S1). This filtering rule resulted in 33 samples with 277 genes involved, and they were subsequently used as our working dataset. The background mutation rate was set as 2.7×10^−6^ for these samples as indicated in the original work [11].

#### Glioblastoma data

The glioblastoma data detected 223 genes with at least one non-silent somatic mutation in one or more samples with experimental validation [3]. A total of 91 samples were examined, including 72 untreated cases and 19 treated cases. To ensure statistical power, we required that a sample would be included for our follow up analysis if it has ≥5 mutated genes. We selected this less stringent cutoff here compared to lung samples due to sample-specific features. As shown in Figure S1, there would be only a few samples remaining if we applied 10 in the glioblastoma data. Thus, using 5 as the cutoff value, 18 samples remained suitable for the following pathway analysis.

As identified in the original work [3], there are 7 hypermutated glioblastoma samples with a high somatic mutation rate, all of which belong to treated samples. These samples resulted in an unequal background mutation rate for treated and untreated samples. Accordingly, we set the mutation rate to be 3.7×10^−6^ for untreated samples and 6.4×10^−6^ for treated samples (http://tcga-data.nci.nih.gov/docs/publications/gbm_2008/TCGA_GBM_Level4_Significant_Genes_by_Mutations_DataFreeze2.xls).

### Weighted resampling based pathway enrichment test in single sample

The underlying assumption of the standard hypergeometric test in gene set enrichment analysis is that all the genes in the genome have an equal chance to be selected. This assumption is no longer valid when the analyzing unit is transferred from mutations to genes, because longer genes tend to have more chances to harbor mutations, assuming the mutations evenly occur across the genome. Thus, the standard hypergeometric test is not applicable in such cases. To this end, we proposed a weighted resampling strategy to build the null distribution, and compared the observed mutated genes in each pathway with the estimated null distribution.

Let *μ* be the background mutation rate for a cancer sample. Previous studies have shown that *μ* is on the order of 10^−6^/nt [12] and varies greatly in different diseases [11], [12]. Here, nt denotes nucleotide. Let *l* be the gene length and *l_i_* for the *i* ^th^ gene, and *G* = {*g_i_*; *i* = 1,…, *n*} be the set of all genes for a total of *n* genes in the genome. Assuming a genomic locus (e.g., nucleotide position) in the genome has two statuses, mutated or not, the probability of the *i* ^th^ gene, *g_i_*, not being mutated could be formulated as *exp*(−*μ×l_i_*) according to the Bernoulli probability, where *exp* is the exponential function. Accordingly, its mutation rate is *m_i_* = 1−*exp*(−*μ×l_i_*). We noted that the estimation of the gene-wise mutation rate could be more complex than simply replying on gene length. Here, we specifically adjusted the gene length bias [7], while a more detailed theorem could be found in literature [12], [13], [14], [15].

**Figure 1:**
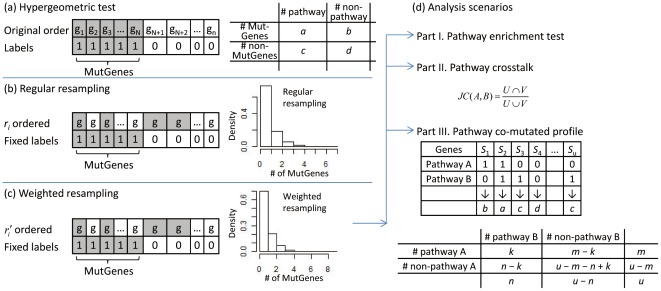
The flowchart of the pathway enrichment pipeline. For a given sample, suppose there are a total of *n* genes in the genome, *G* = {*g_i_*; *i* = 1,…, *n*}, and *N* of them are mutation genes (MutGenes). MutGenes are labeled as 1 while the others are labeled as 0. (a) Hypergeometric test. (b) Regular resampling. (c) Weighted resampling. (d) The three analysis scenarios we performed.

Suppose in an individual genome, a total of *N* genes were detected as mutated among *G* = {*g_i_*; *i* = 1,…, *n*}, and we denote them as “MutGene(s)”, where 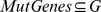. We assign a label for each gene to indicate its mutation status: 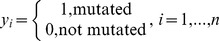 (Figure 1). Given a pathway *S* with *k* MutGenes, our aim is to provide a statistical test to examine whether *S* is significantly enriched with MutGenes. To do so, we can build a null distribution of the MutGenes by randomizing gene labels (Figure 1). Normally, unweighted randomization process assumes every gene has the same chance to be selected as MutGenes. For example, for the *n* genes in *G*, a random number is generated for each of them, i.e., 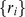, where 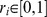 and *i* = 1,…, *n*. Thus, by ordering genes according to their *r_i_* values, gene symbols are randomized while MutGene label, *y_i_*, is fixed (Figure 1b). Repeating this way of permuting gene labels for many times (e.g., 10,000), the background distribution of MutGenes for each pathway can be constructed and the significance of the pathway can subsequently be estimated. This resamping based method of estimating pathway enrichment is complementary to the hypergeometric test, both of which build on the assumption that all the genes have an equal chance to be selected.

In contrast, we proposed the weighted resampling strategy which aims to build the null distribution by projecting each distribution with the same pattern of gene length bias (Figure 1c). Specifically, in each weighted resampling, 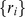 is generated in the same way as in the regular resampling method. However, 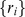 is adjusted for each gene according to the gene-specific mutation rate, i.e., a new random number, 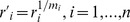, is generated, where 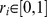 is random numbers and *m_i_* is the gene-wise mutation rate. Genes from *G* are then ordered according to 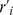. The top *N* genes in the ordered gene list are then assigned as MutGenes for the resample. Note that for longer genes with large values of *m_i_*, 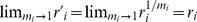, and for shorter genes with small values of *m_i_*, 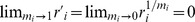. Therefore, for each resample, long genes are more likely to be selected as MutGenes, and these random sets will have the same pattern of gene length as in the real sample. Finally, for each pathway, an empirical *P* value is computed using 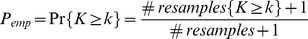, where *k* is the number of MutGenes in the observed case and *K* is the number of “MutGenes” in a resample.

### Pathway crosstalk

We proposed the node-based pathway crosstalk using the *Jaccard coefficient (JC)* measurement, which has been widely applied in set-based analysis [16], [17]. Let *U* indicate the set of genes in pathway A and *V* indicate the set of genes in pathway B, the native *JC* is computed as follows: 
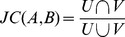
.

To account for the presence of length bias, we also computed *JC* in each weighted resample and computed an empirical *P* value for each pair of pathways as follows: 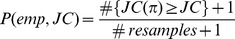, where *JC(π)* is the *JC* value in the *π* ^th^ resampling.

### Co-mutated pathway map

Pathways that are frequently co-mutated across multiple samples could implicate coordinated functions at systems level. To investigate co-mutation events, we first constructed a pathway mutation profile across related samples. As shown in Figure 1d, for each pathway, its mutation status is defined by a binary indicator, i.e., a pathway is indicated as 1 if it is significantly enriched by the weighted resampling strategy; otherwise, 0. For a pair of pathways denoted by *A* and *B*, four categories were proposed to describe the combination pattern of their mutation statuses, i.e., (a) both pathway A and B are significantly enriched, and thus harbor MutGenes, in the same sample, (b) pathway A was significantly enriched, but pathway B was not, (c) pathway B was significantly enriched, but pathway A was not, and (d) neither pathway A nor pathway B was significantly enriched. A 2×2 contingency table was subsequently formulated, and Fisher's exact test was performed to indicate whether the mutation profiles of the two pathways were correlated. Of note, unlike the previous studies which typically counted all pathways that were involved [9], here we only included the significantly enriched pathways identified by our weighted resampling method, as the mutation events in other pathways could be raised by chance.

## Results

### Case study 1: lung adenocarcinomas

#### Pathway enrichment test

**Figure 2:**
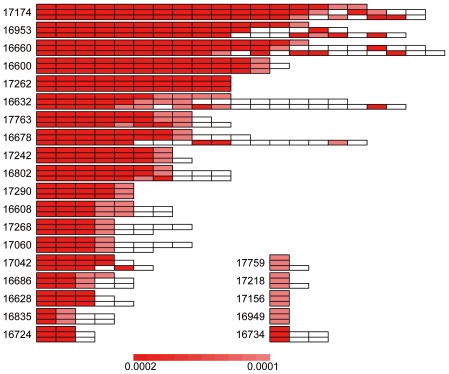
Pathway enrichment test in the lung adenocarcinomas samples. Pathways are represented as rectangles and organized by samples. For each sample, the sample ID is presented on the left and the three rows on the right correspond to results from the weighted resampling method (top row), the regular resampling method (middle row), and hypergeometric test (bottom row), respectively. For each method, the pathways were placed from left to right according to their *P* values with lower *P* values on the left, and, when multiple pathways have the same *P* values, they were ordered by their KEGG ID. To visualize the comparison among methods, each pathway was assigned only one color proportional to its rank in the results from weighted resampling, with darker red implicating lower *P* values. Pathways that are identified by regular resampling or hypergeometric test but not by the weighted resampling are notated in white. Thus, the color of the pathway implicates its rank in the weighted resampling method, and the discordance in the other two rows for a sample shows the different ranking using the other two methods. Note that two samples with the largest number of significantly enriched pathways were not presented in this figure due to space limitations. They are the sample 16668 with 34 significant pathways and the sample 17210 with 22 significant pathways.

For the 33 lung adenocarcinomas samples applicable for pathway enrichment test, the number of MutGenes ranged between 10 and 49, and most (24/33 = 72.72%) were no more than 20 (Figure S1). Using the weighted resampling strategy, 26 samples were identified to have at least one significantly enriched pathway (*P*_Bonferroni_<0.05). As shown in Figure 2, the number of significant pathways varied greatly among samples. The largest number of significant pathways were observed in the sample 16668, with 34 pathways significantly enriched among 38 MutGenes (Table 1), followed by the sample 17210, with 22 significant pathways among 49 MutGenes (data not shown in Figure 2 due to space limitation). Three samples (samples 17174, 16953 and 16660) in the following have 17, 14 and 14 significant pathways, each of which has 13, 16 and 36 MutGenes respectively (Figure 2). Conversely, there are five samples that have only one significant pathways based on the weighted resampling method, while their MutGenes range between 10 and 30, indicating that the number of MutGenes has less influence on the number of significantly enriched pathways in each sample.

**Table 1:** Summary of the sample information and significant pathways (*P*_Bonferroni_<0.05) for the lung adenocarcinomas samples.

Sample ID	# MutGenes	# MutGenes in KEGG	# pathways by weighted resampling	# pathways by regular resampling	# pathways by hypergeometric test
**17210**	49	32	22	27	28
**16668**	38	27	34	32	35
**16660**	36	22	14	20	21
**16835**	30	18	2	4	4
**16686**	29	17	4	5	5
**16678**	28	20	8	11	17
**17759**	24	12	1	1	2
**17290**	23	15	5	5	5
**16628**	22	13	3	4	5
**16632**	20	16	10	16	19
**17218**	17	9	1	1	2
**16953**	16	12	14	16	19
**16608**	13	7	5	7	7
**16734**	13	9	1	3	3
**16802**	13	9	7	10	10
**17174**	13	12	17	20	20
**17262**	13	9	10	10	10
**17268**	12	10	4	8	6
**16600**	11	7	12	13	12
**16724**	11	10	2	3	3
**17060**	11	8	4	8	6
**16949**	10	6	1	1	1
**17042**	10	6	4	5	6
**17156**	10	7	1	1	1
**17242**	10	7	7	7	8
**17763**	10	9	8	9	10

MutGenes: mutation genes.

The most frequently mutated pathways that occurred in more than 10 samples are hsa05220: chronic myeloid leukemia (13/26 samples), hsa05212: pancreatic cancer (12/26 samples), hsa05214: glioma (12/26 samples), hsa05213: endometrial cancer (11/26 samples), hsa05218: melanoma (11/26 samples), and hsa05223: non-small cell lung cancer (11/26 samples). The other lung cancer related pathway, hsa05222: small cell lung cancer, occurred in 3 samples. Table S1 listed the MutGenes that are contributable to the enrichment of these pathways in each of the corresponding samples.

#### Comparison of pathway enrichment methods

As a comparison, we also implemented the standard hypergeometric test and the regular resampling strategy, both of which build on the assumption that all genes have an equal chance of harboring mutations. For the hypergeometric test, the *P* values for each pathway were adjusted by Bonferroni multiple testing correction. For the regular resampling method, the empirical *P* value for each pathway was also adjusted by Bonferroni correction. In all three methods, significant pathways were selected as those with *P*_Bonferroni_<0.05.

We compared the results of the different methods in two ways: the overlapped pathways and the rank of the overlapped pathways. As shown in Figure 2 and Figure S3, approximately two thirds (17 out of 26) of lung adenocarcinomas samples with ≥1 significant pathways have more overlap pathways between the regular resampling method and hypergeometric test than those between regular and weighted resampling methods or those between hypergeometric test and weighted resampling method. In most samples, the pathways identified by the weighted resampling strategy are less than those of regular resampling and standard hypergeometric test (Table 1, Figure S3). Next, we examined the rank of the results using these methods and found that the two resampling based methods showed similar ranking for pathways, while the ranking order of pathways gleaned from the hypergeometric test differs from the other two methods. This is shown by the inconsistency of colors in Figure 2.

Given the difference of the overlap and the rank, we observed that the dissimilarity typically occurred at the end of the pathway list, while the three methods differ only slightly among the most significant pathways. This result indicates that the weighted resampling strategy mainly affects marginally significant pathways, while the pathways with strong evidence of enrichment signals were robust to the gene length bias. This is consistent with a previous work by Wendl et al. [7], who also found that most pathways identified by the standard hypergeometric test did not substantially depart from those identified through unbiased methods, especially for those ranked at the top of the lists. However, the pathways at the bottom of the enrichment lists tend to be false positives, and could only be distinguished when explicitly adjusting the potential biases.

The pathways that are most frequently identified by hypergeometric test but not by weighted resampling include hsa04360: axon guidance (6/26 samples) and hsa05216: thyroid cancer (5/26 samples), followed by hsa04010: MAPK signaling pathway and hsa04012: ErbB signaling pathway in 4 samples, and all the others in less than 4 samples. It is not surprising to see the axon guidance pathway, because it has a large proportion of long genes, and the median gene length of this pathway falls into the upper region of the whole distribution (Figure S2). Similarly, the pathways that are most frequently identified by standard resampling but not by weighted resampling include hsa04360: axon guidance in 5 samples, hsa04010: MAPK signaling pathway in 4 samples, hsa04012: ErbB signaling pathway in 4 samples, and others in less than 4 samples.

#### Pathway crosstalk

**Figure 3:**
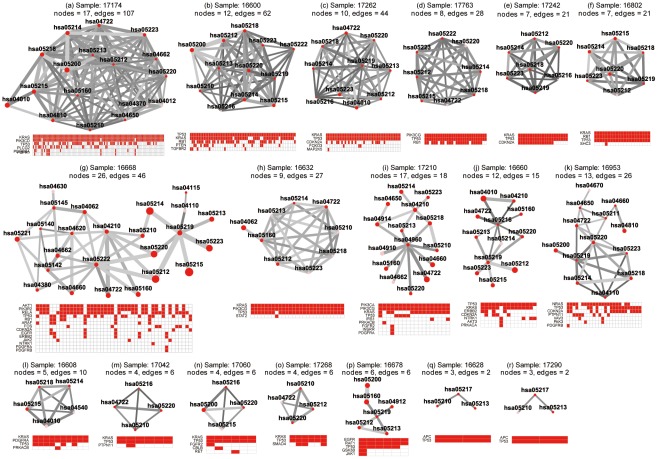
Pathway enrichment map for the lung adenocarcinomas samples. For each sample, the top panel shows the pathway crosstalk map, and the bottom panel shows the genes contributing to the crosstalk. In the top panel, each node represents a pathway with the node color proportional to the pathway enrichment *P* value. The edge represents crosstalk event between the connected nodes (pathways), with edge width proportional to shared MutGenes and edge color proportional to the *P* value of the crosstalk event. In the bottom panel, a matrix shows the profile of genes in the significant pathways, with rows for MutGenes and columns for pathways. When a MutGene is observed in a pathway, the corresponding box is in red.

A total of 18 samples were observed to have at least 2 pathway crosstalk events (*P*_emp_<0.05). We performed multiple testing correction but found no event had *P*_Bonferroni_<0.05. Thus, we selected crosstalk events based on their nominal *P* values, i.e., those with *P*_emp_<0.05. As shown in Figure 3, the crosstalk maps of these 18 samples fell into two major groups: one group with intensive and strong edges among the significant pathways (Figure 3a–3f, 3h, and 3l–3o) and another with sparsely connected networks. Most of the samples in the former group formed cliques or close-to-clique topological units. Here a clique means a fully connected graph in which any two nodes are connected by an undirected edge. In addition, the nominal *P* values of these crosstalk events based on the weighted resampling, as indicated by the darkness of the edges, are typically lower than the later group. The pathways that are frequently involved in this group are mainly related to cancer, such as those with their KEGG ID starting with hsa052XX (X denotes any digit) belonging to the “human diseases→cancers” category in KEGG map [10]. This outcome is not surprising, because in the original definition of pathways in the KEGG database, these cancer pathways share a large proportion of component genes. Further examination of the mutated genes showed that clique-based crosstalk was typically driven by several “hot” MutGenes that participate in multiple cancer-related pathways. For example, the genes *TP53* and *KRAS* co-occur in 11 clique-based crosstalk maps (Figure 3a–3c, 3e, 3f, 3h, 3l–3o), as do other genes such as *RB1*, *PIK3CD*, and *PDGFRA*.

Five samples formed a sparsely connected crosstalk map (Figure 3g, 3i, 3j, 3k, and 3p). Although cancer-related pathways are still the major functional participants in this type of map, there are additional pathways involved, such as hsa04210: apoptosis and hsa04620: toll-like receptor signaling pathway. Investigation of the MutGenes in this type did not show a strong trend toward any gene(s) substantially contributing to the crosstalk events as observed in the clique-group. Finally, two samples displayed the rarest crosstalk events (Figure 3q and 3r), both of which are dominated by the genes *APC* and *TP53*.

#### Pathway co-mutation profile

To explore the co-mutation events that occur among pathways, we started with a list of significantly enriched pathways for each sample (see above). To ensure high quality, pathways that harbored MutGenes but were not significant in a sample were not included for this sample in the co-mutation analysis. As a result, a total of 49 pathways and 26 samples were involved.

**Figure 4:**
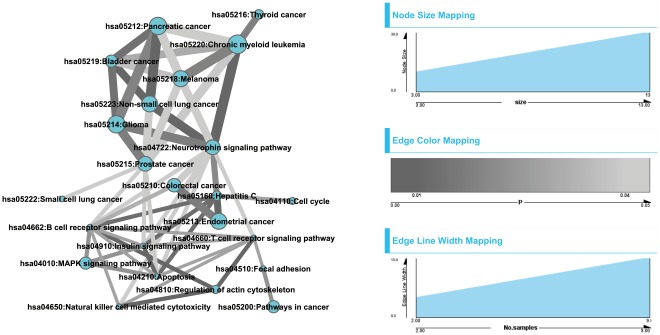
Co-mutation pathway map for the lung adenocarcinomas samples. Node represents pathways that have been identified as significant in at least one sample. An edge between pathways indicates a significant co-mutation event, with edge width proportional to the number of occurring samples of the co-mutation event, and edge color representing the *P* values of the event. Darker edge indicates lower *P* values.

We selected pathways that were co-mutated in 2 or more samples, and had a co-occurrence *P* value that was nominally significant. As shown in Figure 4, two groups were self-clustered, one of which contains several cancer-related pathways, and the other contains several immune-related pathways. In the cancer-related cluster, we observed hsa05214: glioma, hsa05218: melanoma, hsa05219: bladder cancer, hsa05220: chronic myeloid leukemia, and hsa05212: pancreatic cancer. Interestingly, we observed several immune-related pathways in the other cluster, such as hsa04650: natural killer cell mediated cytotoxicity, hsa04660: T cell receptor signaling pathway, hsa04662: B cell receptor signaling pathway, and hsa04210: apoptosis.

### Case study 2: glioblastoma

For the glioblastoma MutGenes, there were a total of 18 samples eligible for the pathway enrichment test (Figure S1), each of which was required to have at least 5 MutGenes. Applying all three methods, i.e., weighted resampling, regular resampling, and the hypergeometric test, we found 15 samples were enriched with at least one pathway by the weighted resampling methods, and these samples were used for the subsequent analysis.

**Figure 5:**
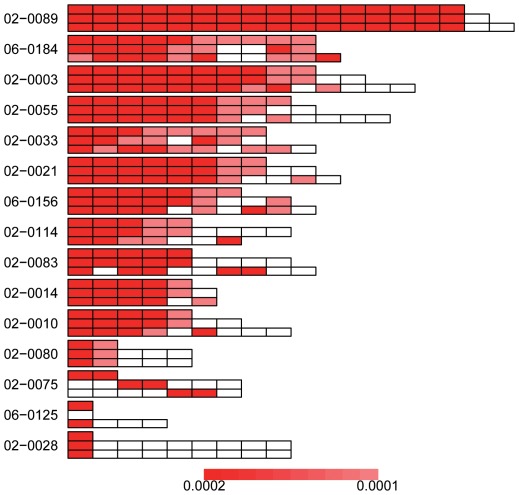
Pathway enrichment test in the glioblastoma samples. Pathways are represented as rectangles and organized by samples. For each sample, the sample ID is presented on the left and the three rows on the right correspond to results from the weighted resampling method (top row), the regular resampling method (middle row), and hypergeometric test (bottom row), respectively. For each method, the pathways were placed from left to right according to their *P* values with lower *P* values on the left, and, when multiple pathways have the same *P* values, they were ordered by their KEGG ID. To visualize the comparison among methods, each pathway was assigned only one color proportional to its rank in the results from weighted resampling, with darker red implicating lower *P* values. Pathways that are identified by regular resampling or hypergeometric test but not by the weighted resampling are notated in white. Thus, the color of the pathway implicates its rank in the weighted resampling method, and the discordance in the other two rows for a sample shows the different ranking using the other two methods.

As shown in Figure 5, the similar trend of pathway overlap and ranking order has been observed in GBM samples as in the lung adenocarcinomas samples. The ranking order between the two resampling methods are closer to each other, and in all the 15 GBM samples the overlapped pathways are found more frequently in the regular resampling method and hypergeometric test than in the weighted resampling results (Figure S4). The most frequently enriched pathways are hsa05200: pathways in cancer (11/15 samples), followed by hsa05214: glioma (9/15 samples), hsa05218: melanoma (9/15 samples), and so on (Figure 5).

**Figure 6:**
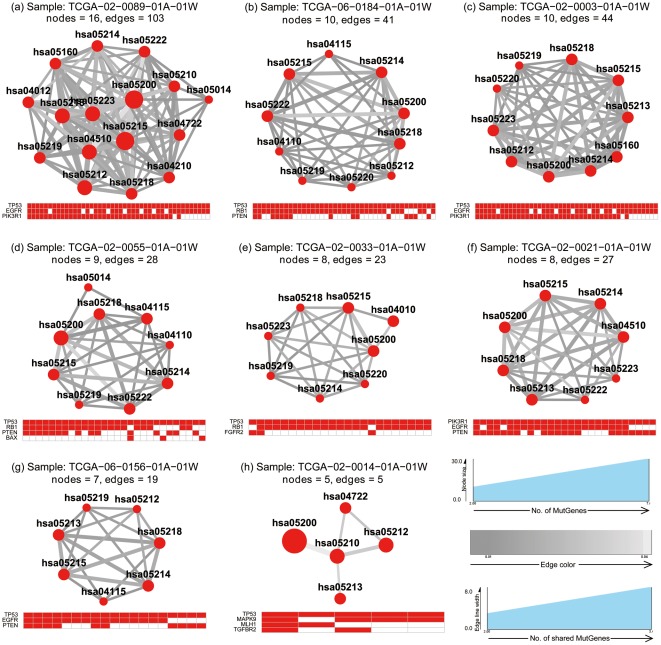
Pathway enrichment map for the glioblastoma samples. For each sample, the top panel shows the pathway crosstalk map, and the bottom panel shows the genes contributing to the crosstalk. In the top panel, each node represents a pathway with the node color proportional to the pathway enrichment *P* value. The edge represents crosstalk event between the connected nodes (pathways), with edge width proportional to shared MutGenes and edge color proportional to the *P* value of the crosstalk event. In the bottom panel, a matrix shows the profile of genes in the significant pathways, with rows for MutGenes and columns for pathways. When a MutGene is observed in a pathway, the corresponding box is in red.

A total of 8 samples were observed to have at least two pathway crosstalk events (Figure 6). Similarly, most of them formed a clique-based topological unit with intensive connections contributed by several “hot” MutGenes such as *TP53*, *EGFR*, *RB1*, *PIK3R1*, and *PTEN* [3], [18].

**Figure 7:**
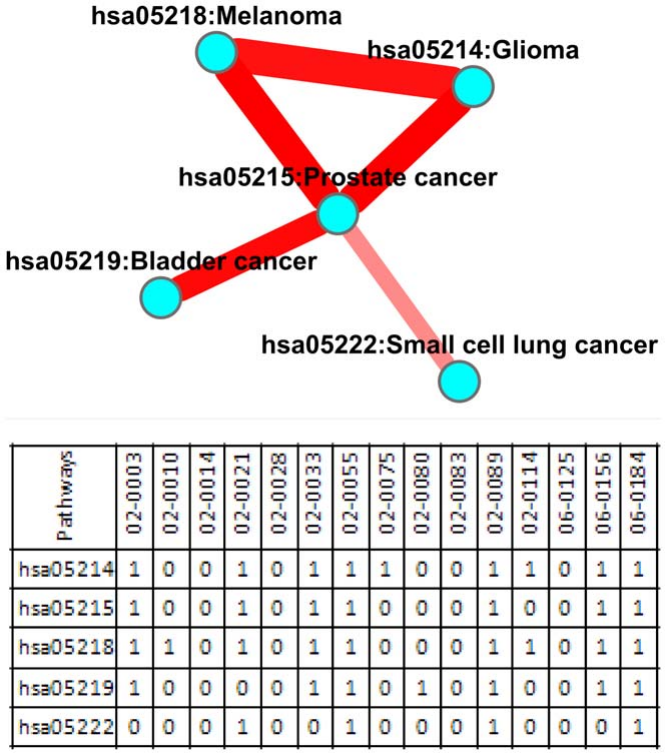
Co-mutation pathway map for the glioblastoma samples. Node represents pathways that have been identified as significant in at least one sample. An edge between pathways indicates a significant co-mutation event, with edge width proportional to the number of occurring samples of the co-mutation event, and edge color representing the *P* values of the event. Darker edge indicates lower *P* values.

Co-mutated pathways in GBM samples are not as prevalent as in lung cancer samples. As shown in Figure 7, there are only five co-mutated events that were observed to be significant (nominal *P* value<0.05, represented by the five edges in Figure 7), involving 5 pathways (represented by the five nodes in Figure 7). They are the pathways of hsa05214: glioma, hsa05218: melanoma, hsa05215: prostate cancer, hsa05219: bladder cancer, and hsa05222: small cell lung cancer. The most frequent co-mutation event was between the pathway of glioma (hsa05214) and prostate cancer (hsa05215), and the pathway of melanoma (hsa05218) and prostate cancer (hsa05215), both of which occurred in 7 samples.

## Discussion

We proposed a bias-reducing strategy in the pathway enrichment test for sample-centered cancer mutations primarily identified from NGS data. By taking the background mutation rate individually, our method performs weighted resampling for pathway enrichment analysis in a sample-specific fashion. Based on this, advanced pathway analyses such as pathway crosstalk as well as pathway co-mutation events could be examined robustly after adjusting the sample-specific mutation rate. Note that our analyses are computationally oriented and with no experimental validation yet. Thus, caution should be taken in interpretation of the results. Although these results are statistically sound, validation in the future work, either by experiments or by other computational approaches, would affirm our findings of driver pathways.

As has been well-studied in previous research, the cancer genome harbors both “driver” mutations, which contribute to the malignancy of cancers, and “passenger” mutations, which have no obvious relationship with the cancerous cells. Many of the passenger mutations are likely by-product events rather than causal factors of the abnormality of the cancer genome, and they could occur randomly in the genome. Due to the limitation of our current knowledge, it is still hard to confidently distinguish driver mutations from passenger mutations, and almost all the current pathway-level analyses use the two types of mutations together. In such a context, it is necessary to adjust potential biases when handling the data to avoid false positive discoveries.

In this study, considering the sample and disease specific mutation characters, we used different cutoff values when selecting lung samples and GBM samples for pathway analysis. Preferably, a cutoff value around 10 to 20 would statistically be more appropriate to ensure the eligibility of the test [19]. In cancer genomes, the mutation rate and mutation patterns differ greatly among various types of cancer, resulting in a great variety of the number of mutation genes among cancer samples. For example, previous studies reported that lung cancer patients typically harbor more mutation genes compared to other types of cancer [20], [21]. In our work, we found a higher cutoff value such as 10 worked reasonably for lung samples, resulting in a selection of an appropriate number of samples for eligible pathway analysis. However, if the same cutoff value (i.e., 10) was applied to GBM samples, it would have only 7 samples after filtering, all of which belong to the treated group, for follow up pathway analysis. As shown in the original work [3], the treated group generally contains more mutations than the untreated group. To make the analysis informative, we subsequently decreased the cutoff value to 5 in GBM sample analysis. This adjustment ensured us to include enough samples from the untreated group.

Our weighted resampling method depends on the gene-wise mutation rate, which relies on two factors, the sample-specific background mutation rate *μ* and the particular gene length *l*. Inclusion of *μ* ensures that when comparing different individual samples, and even different diseases, the analysis can be built on the same scale. Thus for cross-sample analysis, such as the pathway co-mutation map, the sample-specific rate *μ* is particularly important. However, for a given sample in which *μ* is fixed, the gene-wise mutation rate will mainly behave as a function of gene length. Our weighted resampling method explicitly incorporates gene-wise mutation rate in order to build the gene-length matched null distribution for each pathway. Thus, the empirical *P* values obtained in this way can overcome the length bias and provide more accurate statistic evaluation of the enrichment signals.

The estimation of the background mutation rate in most studies adopted the formula *μ* = *ρ_NS_* = *ρ_S_*×*R*, where *ρ_NS_* is the nonsynonymous (NS) mutation rate, *ρ_S_* is the synonymous (S) mutation rate, and *R* is the ratio of NS to S substitutions [11], [12], [13], [15]. *ρ_S_* can be directly computed by *ρ_S_* = {#synonymous}/{#sequenced base pairs}, and *R* can be estimated through a random mutation model that takes into consideration nucleotide composition in the coding regions [22], [23]. In our study, we used the somatic mutation data for lung and GBM samples which were initially detected through targeted sequencing in pre-selected genes. Although in each study, hundreds of genes were sequenced, they still did not reach the genome-wide scale and the number of NS/S mutations was limited in each sample. The original studies for these two datasets estimated the mutation rate by collapsing all samples [11], or by sub-groups of samples [3], rather than computing individual level mutation rate. In future studies, since mutation data from whole genome sequencing and whole exome sequencing will be easily obtained for individuals [24], our method will find more applications by using these data for mutation background estimation.

As shown in the lung cancer sample, there were indeed several pathways with long genes filtered out by the weighted resampling strategy, while they were included in both regular resampling and the hypergeometric test. However, the difference among the three methods we tested seems to only affect marginally significant pathways, i.e., pathways that are ranked at the end of the resultant pathway list by each method (Figure 2). This is not surprising, as the length bias may not be substantially significant in all samples, and the sequenced genes we used in this study are limited to pre-defined candidate genes rather than genome-wide sequencing. For example, the lung cancer data was collected from a total of 623 candidate genes consisting of oncogenes, tumor suppressor genes, genes from protein kinase families, and others [11], and the glioblastoma data was from sequencing of 601 pre-selected genes [3]. Nevertheless, in both cases, there are pathways that were identified as significant through standard statistical tests (e.g., hypergeometric test or the regular resampling method) but were found to be non-significant by the weighted resampling strategy after considering gene length bias. Importantly, most of these error-prone pathways contain long MutGenes. An example is the pathway of axon guidance (hsa04360) in 6 lung cancer samples. The rejection of these long gene pathways by the weighted resampling method shows the rationality of reducing length bias.

It is important to recognize the long gene effect, and the possibility of false discovery using methods without length bias adjustment. For example, Gu et al. [9] analyzed the co-mutation profile of pathways in the same lung adenocarcinomas data as we used here. However, without appropriate adjustment of gene length, several pathways that are known to harbor long genes were included in their co-mutated pathway network, such as axon guidance, long-term potentiation, and long-term depression [9]. These pathways function in neuro- and brain-systems, but there is rare evidence showing their functions related to cancer. We provided an updated co-mutation profile using the significant pathways resulting from the weighted resampling strategy, effectively removing these possible false discoveries.

The gene length problem not only exists in mutation data, but many other types of data where there is a necessary step to map an original analysis unit (e.g., mutated locus, SNP, CNV, methylation locus) to the unit of gene (i.e., the unit for pathway organization). For example, in pathway enrichment analyses of genome-wide association studies, in which millions of single nucleotide polymorphisms (SNPs) are genotyped using microarray chips, the step of mapping SNPs to genes could generate more SNPs for long genes and, thus, long genes would have a higher probability to be significant. Another example is CNV data, where there is a similar problem in that long genes tend to be more frequently affected by CNV regions [25] and require careful adjustment before any conclusion is made. Thus, the weighted resampling strategy we proposed in this study for cancer mutation data could be easily extended to other types of genetic mutations and widely applied to different kinds of diseases.

In summary, we proposed a weighted resampling strategy to adjust gene length bias in pathway enrichment analysis of a set of cancer genes. This strategy incorporates gene mutation rates and is implemented in a sample-specific way, thus enabling the identification of a personalized pathway enrichment map. Demonstrated in two cancer projects, the weighted resampling strategy could effectively reduce the burden of long gene pathways and provide a complementary method in the field.

## Supporting Information

Figure S1 **Distribution of the number of mutation genes in the lung adenocarcinomas dataset (a) and in the glioblastoma dataset (b).** The horizontal dash lines indicate the cutoff values we applied to select samples with appropriate number of mutation genes for pathway analysis. The vertical dash lines indicate that the samples on the right of the lines were selected.
(PDF)

Figure S2 **Distribution of the gene length for KEGG pathways.** We used the median values of the gene lengths for each pathway. The red line indicates the pathway hsa04360: axon guidance.
(PDF)

Figure S3 **Venn diagrams showing the overlap pathways identified in each lung adenocarcinomas sample by three methods: hypergeometric test (hyper), regular resampling (regular), and weighted resampling (weighted).**
(PNG)

Figure S4 **Venn diagrams showing the overlap pathways identified in each glioblastoma sample by three methods: hypergeometric test (hyper), regular resampling (regular), and weighted resampling (weighted).**
(PNG)

Table S1 The most frequently mutated pathways (>10 samples) in lung adenocarcinomas data.
(DOCX)
