# Supplementary figures and images for: Personalized Pathway Enrichment Map of Putative Cancer Genes from Next Generation Sequencing Data
Source: PLoS One. 2012 May 18;7(5):e37595. doi: 10.1371/journal.pone.0037595 (PMC3356304; doi:10.1371/journal.pone.0037595)

**Lung adenocarcinomas samples**

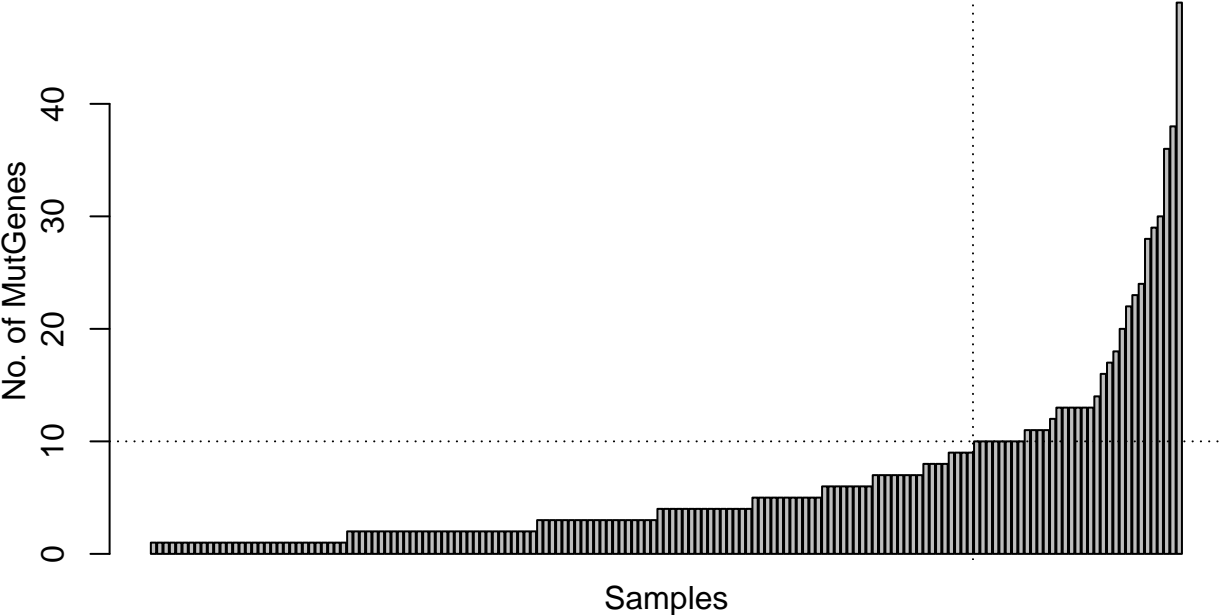

**Glioblastome cancer samples**

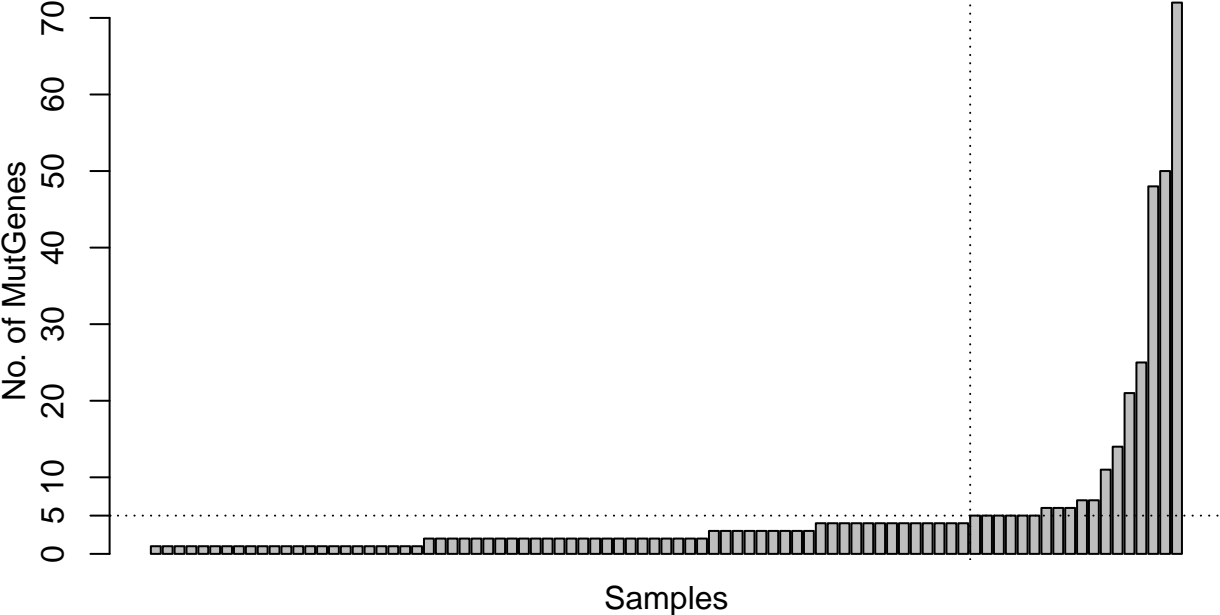

Supplement: Figure S1 — Distribution of the number of mutation genes in the lung adenocarcinomas dataset (a) and in the glioblastoma dataset (b). The horizontal dash lines indicate the cutoff values we applied to select samples with appropriate number of mutation genes for pathway analysis. The vertical dash lines indicate that the samples on the right of the lines were selected. (PDF) [file pone.0037595.s001.pdf]

**Distribution of gene length for KEGG pathways**

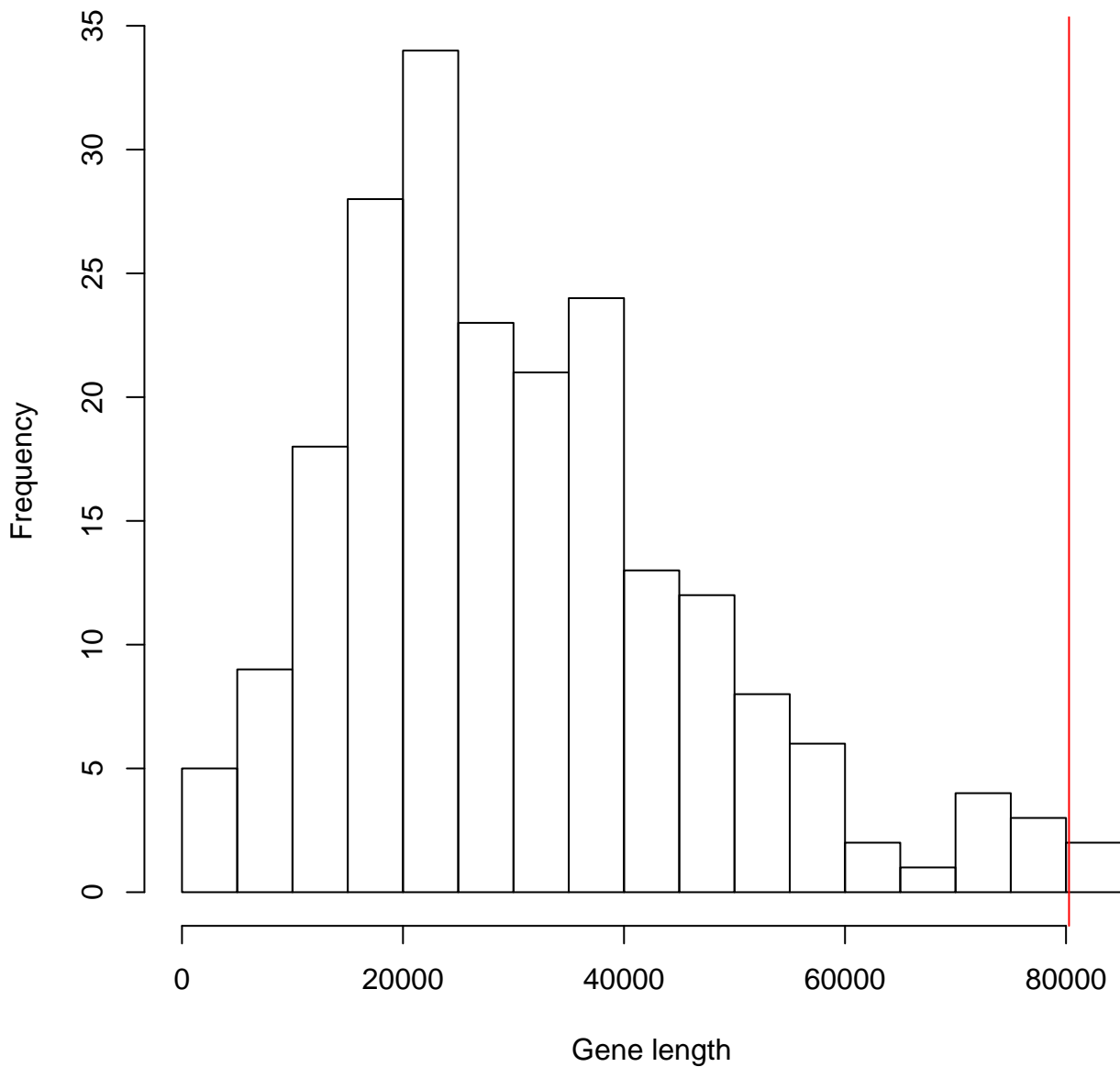

Supplement: Figure S2 — Distribution of the gene length for KEGG pathways. We used the median values of the gene lengths for each pathway. The red line indicates the pathway hsa04360: axon guidance. (PDF) [file pone.0037595.s002.pdf]

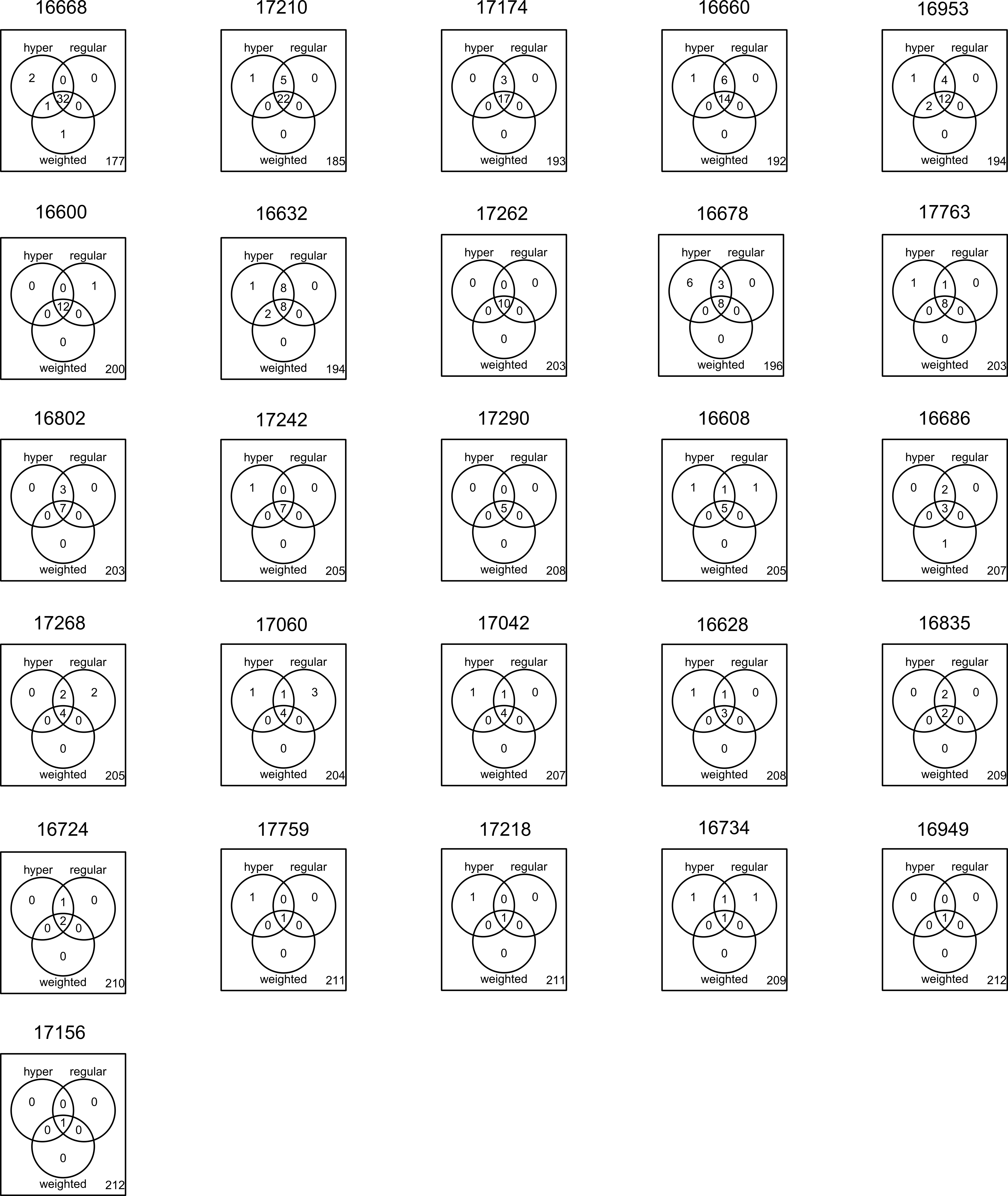

Supplement: Figure S3 — Venn diagrams showing the overlap pathways identified in each lung adenocarcinomas sample by three methods: hypergeometric test (hyper), regular resampling (regular), and weighted resampling (weighted). (PNG) [file pone.0037595.s003.png]

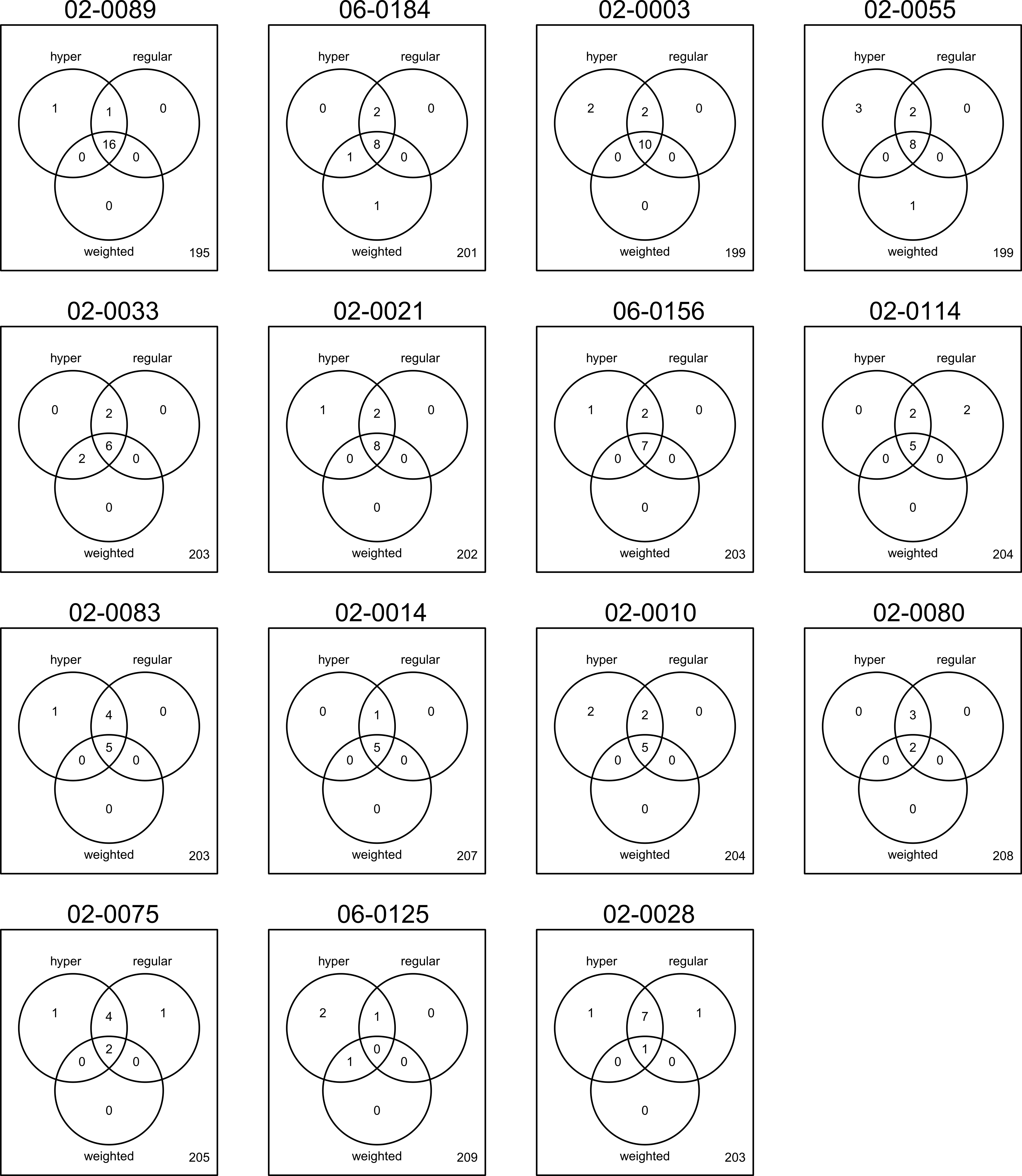

Supplement: Figure S4 — Venn diagrams showing the overlap pathways identified in each glioblastoma sample by three methods: hypergeometric test (hyper), regular resampling (regular), and weighted resampling (weighted). (PNG) [file pone.0037595.s004.png]
