# Supplementary material for: Personalized Pathway Enrichment Map of Putative Cancer Genes from Next Generation Sequencing Data
Source: PLoS One. 2012 May 18;7(5):e37595. doi: 10.1371/journal.pone.0037595 (PMC3356304; doi:10.1371/journal.pone.0037595)
Supplement: Table S1 — The most frequently mutated pathways (>10 samples) in lung adenocarcinomas data. (DOCX) [file pone.0037595.s005.docx]

**Table S1.** The most frequently mutated pathways (>10 samples) in lung adenocarcinomas data. MutGenes: mutation genes.

| **Pathway** | **Sample ID: MutGenes** |
| --- | --- |
| hsa05220 | Sample 16600: KRAS, RB1, TGFBR2, TP53 |
|  | Sample 16632: KRAS, PIK3CG, TP53 |
|  | Sample 16660: AKT3, CDKN2A, KRAS, TP53 |
|  | Sample 16668: CDKN2A, AKT1, ARAF, PIK3R2, RB1, RELA, STAT5A, TP53 |
|  | Sample 16802: KRAS, SHC3, RB1, TP53 |
|  | Sample 16953: CDKN2A, NRAS, PTPN11, TP53 |
|  | Sample 17042: KRAS, PTPN11, TP53 |
|  | Sample 17060: KRAS, TP53, CBLB |
|  | Sample 17174: KRAS, PIK3CD, TP53 |
|  | Sample 17210: KRAS, PIK3CA, PIK3CG, TP53 |
|  | Sample 17242: CDKN2A, KRAS, TP53 |
|  | Sample 17262: CDKN2A, KRAS, TP53 |
|  | Sample 17268: KRAS, SMAD4, TP53 |
|  | Sample 17763: PIK3CG, RB1, TP53 |
| hsa05212 | Sample 16600: KRAS, RB1, TGFBR2, TP53 |
|  | Sample 16632: KRAS, PIK3CG, RALB, TP53 |
|  | Sample 16660: AKT3, CDKN2A, ERBB2, JAK1, KRAS, RALA, TP53 |
|  | Sample 16668: CDKN2A, EGFR, ERBB2, AKT1, ARAF, PIK3R2, RB1, RELA, TP53 |
|  | Sample 16678: EGFR, JAK1, RAF1, TP53 |
|  | Sample 16686: SMAD4, MAPK8, BRCA2 |
|  | Sample 16802: KRAS, RB1, TP53 |
|  | Sample 17174: KRAS, PIK3CD, TP53 |
|  | Sample 17210: KRAS, PIK3CA, PIK3CG, TP53 |
|  | Sample 17242: CDKN2A, KRAS, TP53 |
|  | Sample 17262: CDKN2A, KRAS, TP53 |
|  | Sample 17268: KRAS, SMAD4, TP53 |
|  | Sample 17759: BRAF, BRCA2, TP53 |
|  | Sample 17763: PIK3CG, RB1, TP53 |
| hsa05214 | Sample 16600: KRAS, PTEN, RB1, TP53 |
|  | Sample 16608: KRAS, PDGFRA, TP53 |
|  | Sample 16632: KRAS, PIK3CG, TP53 |
|  | Sample 16660: AKT3, CDKN2A, KRAS, TP53 |
|  | Sample 16668: CDKN2A, EGFR, AKT1, ARAF, PDGFRA, PDGFRB, PIK3R2, RB1, TP53 |
|  | Sample 16802: KRAS, SHC3, RB1, TP53 |
|  | Sample 16953: CDKN2A, NRAS, PDGFRB, TP53 |
|  | Sample 17174: KRAS, PDGFRA, PIK3CD, PLCG2, TP53 |
|  | Sample 17210: KRAS, PDGFRA, PIK3CA, PIK3CG, TP53 |
|  | Sample 17242: CDKN2A, KRAS, TP53 |
|  | Sample 17262: CDKN2A, KRAS, TP53 |
|  | Sample 17763: PIK3CG, RB1, TP53 |
| hsa05213 | Sample 16600: KRAS, PTEN, TP53 |
|  | Sample 16628: APC, KRAS, TP53 |
|  | Sample 16632: KRAS, PIK3CG, TP53 |
|  | Sample 16660: AKT3, ERBB2, KRAS, TP53 |
|  | Sample 16668: EGFR, ERBB2, AKT1, ARAF, PIK3R2, TP53 |
|  | Sample 16678: EGFR, GSK3B, RAF1, TP53 |
|  | Sample 17060: KRAS, TP53 |
|  | Sample 17174: KRAS, PIK3CD, TP53 |
|  | Sample 17210: KRAS, PIK3CA, PIK3CG, TP53 |
|  | Sample 17218: EGFR, APC, TP53 |
|  | Sample 17262: FOXO3, KRAS, TP53 |
|  | Sample 17290: APC, MYC, TP53 |
| hsa05218 | Sample 16600: KRAS, PTEN, RB1, TP53 |
|  | Sample 16608: KRAS, PDGFRA, TP53 |
|  | Sample 16660: AKT3, CDKN2A, KRAS, TP53 |
|  | Sample 16668: CDKN2A, EGFR, AKT1, ARAF, PDGFRA, PDGFRB, PIK3R2, RB1, TP53 |
|  | Sample 16802: KRAS, RB1, TP53 |
|  | Sample 16953: CDKN2A, NRAS, PDGFRB, TP53 |
|  | Sample 17174: FGFR1, KRAS, PDGFRA, PIK3CD, TP53 |
|  | Sample 17210: KRAS, PDGFRA, PIK3CA, PIK3CG, TP53 |
|  | Sample 17242: CDKN2A, KRAS, TP53 |
|  | Sample 17262: CDKN2A, KRAS, TP53 |
|  | Sample 17763: PIK3CG, RB1, TP53 |
| hsa05223 | Sample 16600: KRAS, RB1, TP53 |
|  | Sample 16632: KRAS, PIK3CG, TP53 |
|  | Sample 16660: AKT3, CDKN2A, ERBB2, KRAS, TP53 |
|  | Sample 16668: CDKN2A, EGFR, ERBB2, AKT1, ARAF, PIK3R2, RB1, TP53 |
|  | Sample 16802: KRAS, RB1, TP53 |
|  | Sample 16835: FOXO3, PRKCG, TP53 |
|  | Sample 16953: CDKN2A, NRAS, TP53 |
|  | Sample 17060: KRAS, TP53 |
|  | Sample 17174: KRAS, PIK3CD, PLCG2, TP53 |
|  | Sample 17210: KRAS, PIK3CA, PIK3CG, TP53 |
|  | Sample 17242: CDKN2A, KRAS, TP53 |
|  | Sample 17262: CDKN2A, FOXO3, KRAS, TP53 |
|  | Sample 17763: PIK3CG, RB1, TP53 |
